# Analysis of the effects of the age-period-birth cohort on cervical cancer mortality in the Brazilian Northeast

**DOI:** 10.1371/journal.pone.0226258

**Published:** 2020-02-19

**Authors:** Karina Cardoso Meira, Glauber Weder dos Santos Silva, Juliano dos Santos, Raphael Mendonça Guimarães, Dyego Leandro Bezerra de Souza, Gilcilene Pretta Cani Ribeiro, Eder Samuel Oliveira Dantas, Jovanka Bittencourt Leite de Carvalho, Rafael Tavares Jomar, Taynãna César Simões

**Affiliations:** 1 Health School, Federal University of Rio Grande do Norte, Natal, Rio Grande do Norte, Brazil; 2 Department of Nursing, Federal University of Rio Grande do Norte, Natal, Rio Grande do Norte, Brazil; 3 Cancer Hospital III, National Cancer Institute, Rio de Janeiro, Rio de Janeiro, Brazil; 4 Joaquim Venâncio Polytechnic School, Oswaldo Cruz Foundation, Rio de Janeiro, Rio de Janeiro, Brazil; 5 Collective Health Department, Federal University of Rio Grande do Norte, Natal, Rio Grande do Norte, Brazil; 6 Biologist, specialist in management in Health Systems and Services, State Secretariat of Espírito Santo, Vitória, Espírito Santo, Brazil; 7 Assistance Coordination, National Cancer Institute, Rio de Janeiro, Rio de Janeiro, Brazil; 8 René Rachou Institute, Oswaldo Cruz Foundation, Belo Horizonte, Minas Gerais, Brazil; London School of Hygiene and Tropical Medicine, UNITED KINGDOM

## Abstract

Cervical cancer (CC) is a public health problem with a high disease burden and mortality in developing countries. In Brazil, areas with low human development index have the highest incidence rates of Brazil and upward temporal trend for this disease. The Northeast region has the second highest incidence of cervical cancer (20.47 new cases / 100,000 women). In this region, the mortality rates are similar to rates in countries that do not have a health system with a universal access screening program, as in Brazil. Thus, this study aimed to analyze the effects of age, period and birth cohorts on mortality from cervical cancer in the Northeast region of Brazil. Estimable functions predicted the effects of age, period and birth cohort. The average mortality rate was 10.35 deaths per 100,000 women during the period analyzed (1980–2014). The highest mortality rate per 100,000 women was observed in Maranhão (24.39 deaths), and the lowest mortality rate was observed in Bahia (11.24 deaths). According to the period effects, only the state of Rio Grande do Norte showed a reduction in mortality risk in the five years of the 2000s. There was a reduction in mortality risk for birth cohorts of women after the 1950s, except in Maranhão State, which showed an increasing trend in mortality risk for younger generations. We found that the high rates of cervical cancer mortality in the states of northeastern Brazil remain constant over time. Even after an increase in access to health services in the 2000s, associated with increased access to the cancer care network, which includes early detection (Pap Test), cervical cancer treatment and palliative care. However, it is important to note that the decreased risk of death and the mortality rates from CC among women born after the 1960s may be correlated with increased screening coverage, as well as increased access to health services for cancer treatment observed in younger women.

## Introduction

Cervical cancer (CC) represents the fourth most common cancer in women worldwide. In addition, more than 90% of deaths occur in developing countries, where women are 18 times more likely to die from this cancer than women in developed countries [1]. CC is considered a neglected neoplasm because it is based on early detection by screening methods for Pap Test (oncotic cytology), which allows the diagnosis of the disease in its incipient and curable phase [2–4]. These methods have been correlated with reduced incidence and mortality from CC in developed countries, with organized programs of high coverage and quality [5–10]. However, when there is a CC prevention and control program in developing countries, it is carried out opportunistically, with poor coverage and quality, with inadequate funding and an insufficient cancer care network [1,2–11].

Brazil has a population of approximately 207.7 million. The country is divided into 26 states and one federal government. The Federation is further grouped into five major regions (North, Northeast, Southeast, South and Midwest) with different geographical, economic and cultural characteristics. The best human development indexes are observed in the South. The Southeast, the most populous region, stands out for its labor market. Although the Midwest includes the nation’s capital, it has an economy focused on agriculture and livestock. The Northeast region has the lowest rates of human development, and difficult access to health, education and sanitation services, representing a place of high socioeconomic vulnerability, and its territory is divided into nine states: Alagoas, Bahia, Ceará, Maranhão, Paraíba, Pernambuco, Piauí, Rio Grande do Norte and Sergipe. Finally, the North has the second worst development rates in the country and is characterized by its low population density due to the Amazon rainforest.

The National Screening Program had its guidelines implemented in 1999 in the National Cervical Cancer Control Program (*PNCC*), but since the 1980s, the states of the South and Southeast, the most developed regions of Brazil, have implemented screening based on the Pap test [2]. These states showed a significant reduction in cervical cancer mortality in the late 1990s and 2000s. In addition, more than 50% of the Brazilian cancer care network is concentrated in these regions; therefore, greater access to secondary prevention and treatment may be correlated with the reduced risk of death in these states, even among generations of women with greater exposure to risk factors [12,13].

The temporal trend of cervical cancer mortality depends on several different factors and may be correlated with the prevalence of risk factors in the female population, access to the screening program, and cancer care services for surgery, chemotherapy, and radiotherapy [1,2–11]. The existence of an organized screening program associated with a universal access cancer care network enables early diagnosis and timely treatment of this disease, and can therefore be correlated with lower incidence and mortality from cervical cancer [1,2–11].

Thus, it is important to highlight that the Brazilian Cervical Cancer Program corresponds to free and universal access to the prevention and treatment of cervical cancer. This program includes primary health care (screening), referral for treatment of CC precursor lesions, cancer treatment in specialized hospitals (surgery, radiotherapy, chemotherapy and palliative care) [2,12–15]. Therefore, the analysis of the temporal trend of mortality from this cancer is an important tool to raise hypotheses about the efficacy and effectiveness of *PNCC*.

Following the implementation of the PNCC in Brazil, an increase in screening coverage was observed in all Brazilian regions due to increased access to primary health care [14]. However, there are significant disparities in the coverage and quality of oncological cytological examination according to geographic regions, area of residence (urban or rural), and sociodemographic characteristics of women [14,16–17].

In all Brazilian regions and states, the lowest coverage was observed in women aged 50 years over, black, with up to 4 years of education, living in the Northeast and North states and who did not have private health insurance [14,16–17]. In addition, the cancer treatment network is concentrated in the regions with the highest socioeconomic development in the country (South and Southeast), and women living in the North and Northeast must travel long distances to access cancer treatment [12,13]. This reality possibly correlates with the maintenance of the high incidence and mortality rates of CC in Brazilian regions with the worst socioeconomic indicators [18–21].

The estimate of cervical cancer in Brazil in 2018/2019 shows that this cancer in women is the most incident in the North region (25.62/100,000 women), and the second most incident in the Northeast region (20.47 deaths/100,000 women), excluding non-melanoma skin cancer [22], while in the more developed regions of the country (in the South and Southeast regions), cervical cancer represents the third most incident cancer, being surpassed by breast cancer and colorectal cancer.

In addition, an increasing mortality from this neoplasm was found in the interior regions of the North and Northeast from 1980 to 2005, while the temporal trend was decreasing in the capitals and interior regions in the South and Southeast regions [18–20], a profile that was maintained in more recent studies [21,23].

The northeastern and northern states of Brazil have incidence and mortality rates similar to those of countries without a universal health system, screening program and free cancer care network [1–3], showing the alarming situation that occurs in this Brazilian region.

A study carried out from 1996 to 2005 in municipalities of the Northeast region indicated higher mortality rates of cervical cancer in the interior cities. In addition, a positive correlation was found between CC mortality and socioeconomic factors that indicate high socioeconomic vulnerability, such as the proportion of illiterate 25 years or older, high fertility rate, the proportion of inhabitants below the poverty line (< minimum half per capita salary), and child mortality (up to 5 years per 1,000 live births) [19].

This reality remained due to the structural heterogeneity in which there is a progressive expansion of socioeconomic inequalities between the richest and poorest regions of developing countries [24]. This has an impact on the morbidity and mortality profile; thus, the most developed regions have higher incidence rates and cancer mortality associated with population aging and westernized habits and lifestyle, while the poorer regions have higher magnitude of neoplasms associated with infections [1,5].

Age, period and birth cohort are factors that influence the temporal trend of incidence and mortality of diseases and health injuries. The age effects are changes associated with chronological age, which may arise from physiological changes, accumulation of socioeconomic, cultural experiences or a combination of these over a lifetime [25–33]. These age-related changes may increase or decrease the risk of disease and/or death [25–33]. Thus, younger individuals are at greater risk of illness and death from sexually transmitted infections and external causes (homicides and traffic accidents); On the other hand, older individuals are at greater risk of becoming ill and dying from noncommunicable diseases. Therefore, the age structure of a population interferes with the incidence and prevalence of noncommunicable diseases and violence [25–33].

The period effect refers to structural changes that affect all age groups simultaneously, corresponds to a complex set of historical, socioeconomic and cultural events and environmental factors [25–32] such as world wars, expansion or economic crisis, pandemic and epidemic of certain infections, Public Health policies, therapeutic innovations and expanding access to health services [33]. These factors that occur at specific times can change incidence, prevalence and mortality rates across all age groups simultaneously [33].

Another factor worth mentioning is the cohort effect, as individuals from the same cohort undergo socioeconomic, cultural and environmental changes at the same age. Thus, members of different birth cohorts will be exposed differently throughout life to risk and protective factors for diseases and health problems, interfering with the difference in the risk of disease and death between different birth cohorts [25–33].

The temporal trend of cervical cancer incidence and mortality rates may be correlated with: the prevalence of exposure to disease risk factors in the female population (early onset of sexual activity, increased number of sexual partners in women or men with a large number of female sexual partners, contraceptive use, smoking, high parity, and diseases that reduce immunity in women, among others), the presence, quality and coverage of a screening program; and access to cancer care network for timely treatment of the disease (surgery, chemotherapy and radiotherapy) [2,10].

It is noteworthy that reduction in cervical cancer mortality in recent decades in several countries around the world was correlated with access to screening, which reduced the incidence of the disease through the identification of cases and consequent treatment of precursor lesions, considering that, in recent decades, there have been no significant changes in the treatment of advanced disease [3–11].

However, we believe that even with increased access to health services in the 2000s, including a free access screening program (PNCC) and cancer care network, there has been no reduction in cervical cancer mortality in the Northeast states, due to their high socioeconomic vulnerability.

Pap Test coverage among women living in the Northeast states is lower than women living in more developed regions of the country (South and Southeast) [16–17], and this region has the lowest proportion of the cancer care network in the country [22]. However, there may be a reduction in the risk of death in specific birth cohorts, because access to health services and Pap test coverage are differentiated between birth cohorts and higher in younger cohorts [14–17].

Most Brazilian studies evaluating cervical cancer mortality performed analyses summarized by age group and death period, disregarding the cohort effect [18–23], an extremely important factor in the evaluation of temporal trend the incidence and mortality from noncommunicable chronic diseases [25–33].

Cervical cancer has its incidence and mortality influenced by the prevalence of its risk and protective factors in the female population. The prevalence of exposure to these factors has disparities between different generations of women, which may have a cohort effect on the incidence and mortality of this cancer. This difference can be captured by an analysis of the effects of age, period and cohort Thus, this study aims to analyze the effects of age, period and birth cohorts on cervical cancer mortality in the Northeast region of Brazil.

## Materials and methods

### Study design and population

This ecological study evaluated cervical cancer mortality in the Northeast (NE) states of Brazil from 1980 to 2014. The NE occupies an area of 1,554,291.6 km^2^, with an estimated population from 2019 to 57,883,049 inhabitants, covering nine federal units: Alagoas, Bahia, Ceará, Maranhão, Paraíba, Pernambuco, Piauí, Rio Grande do Norte and Sergipe. This area has the lowest socioeconomic development in the country, with high fertility and child mortality rates and a higher Gini index [34,35].

Studies have shown that regions with low socioeconomic development have higher rates of cancer incidence and mortality associated with infection [1,5]. In this context, in Brazil, there was a positive correlation between cervical cancer mortality and the municipalities with the worst socioeconomic indicators [36]. Thus, in the present study we analyzed whether there was a reduction in the risk of death from cervical cancer in the Northeast states, in the periods after the implementation of PNCC, especially in the younger cohorts, which present greater cervical cancer screening and access to health services. The PNCC was implemented in the late 1990s, representing the free and universal cervical cancer care network, including early detection (Pap Test), treatment (surgery, radiotherapy and chemotherapy) and palliative care.

The data used in this study were freely accessed from the Mortality Information System of the Informatics Department of the Unified Health System (*SIM/DATASUS*) on the website: http://www2.datasus.gov.br/DATASUS/ [35]. There are no identified individuals in this system; therefore, this study was not submitted to a Research Ethics Committee.

The *SIM/DATASUS* contains data available for free download on mortality from 1979 to 2016 [35]. It is noteworth that mortality data for Brazil are available until 2016, but we chose to work with age groups and periods of the same size to avoid an identifiable problem due to artificial cyclical patterns when using different periods and age ranges in the estimate [25–30]. The Ninth International Classification of Diseases (ICD-9) operated from 1979 to 1995, while the Tenth International Classification of Diseases (ICD-10) entered into force from 1996 to 2016.

Population data for mortality rate estimates were also obtained from *DATASUS*, based on a demographic census from 1980, 1991, 2000 and 2010. The Brazilian Institute of Geography and Statistics estimated populations projections on July 10 of the intercensal years [34].

### Study variables

The following Ninth and Tenth International Classification of Diseases (ICD-9 and ICD-10) classifications were taken from *SIM/DATASUS*: cervical cancer (CC): 180 (ICD-9) and C53 (ICD-10); incomplete diagnosis of general cancer and incomplete diagnosis of female genital tract cancer (184, 195, 196, 197, 198, 199, C57, C76, C77, C78, C79, C79, C97, C76, C77, C80); and nonspecific portion uterine cancer(NSP, 179 and C55) [35].

The quality of information and coverage of *SIM/DATASUS* death records has improved in recent decades; However, there are significant disparities between regions according to socioeconomic development. In fact, between the 1990s and 2000s, there was a significant improvement in the information coverage and quality for all geographic regions of Brazil [19,37–40]. However, states in the northern and northeastern regions with the lowest socioeconomic development still present significant problems in their Mortality Information Systems [37–40].

For this reason, Brazilian authors do not recommend studies of temporal trends of geographic regions based on raw data from the Mortality Information System (*SIM/DATASUS*), suggesting the use of indirect techniques in a five-step procedure to correct the death records [18–20].

Considering the problems associated with the coverage and quality of death registrations in Brazil and the long period of analysis in the present study for the International Classifications of Diseases (ICD-9 and ICD-10), techniques were applied to correct these limitations [19,38,40,41].

The correction process was independently carried out by three authors, confirmed by a fourth author, and included the following steps: (i) proportional redistribution of 50% of deaths classified as ill-defined cause among defined natural causes [20,41], stratified by the northeast states; (ii) the proportional redistribution, according to age group and year, of deaths classified as incomplete diagnosis among all cancers; the proportional redistribution, by age group and year, of deaths classified as incomplete diagnosis of female genital tract cancer, stratified by northeastern states; (iii) the proportional redistribution, according to age group and year, of deaths classified as unspecified uterine cancer; (iv) the sum of the values obtained in the previous steps was added to the cervical cancer deaths registered in SIM/DATASUS; and (v) finally, a correction in death coverage (underreporting), using the correction factors proposed by Queiroz et al. (2017) [40], for females according to the Brazilian states of the 1980s, 1990s, 2000s and 2010s. At this stage, the correction factors for each decade were multiplied by the number of deaths obtained in step iv.

When correcting the death records, we chose to work with age groups and periods grouped at five-year intervals. Age groups from 20–24 years to 80 years or older were evaluated due to excess zeros in smaller groups, resulting in *I* = 13 age groups, *J* = 7 time periods, and *K* = *I* + *J*– 1 = 19 birth cohorts, ranging from 1900 to 1990 [24–25]. Where i = 1, …., I; j = 1, … J; k = 1, …, K; and where K = I + J-1. It is highlighted that the year and age groups proportionally redistributed ignored age groups.

Cervical cancer mortality rates, age group and geographic region per 100,000 women were calculated by 5-year age groups. Truncated rates for ages at open intervals (80 years and over) were calculated by year. After obtaining the rates by age groups and open ages intervals, the five-year periods were standardized by the direct method, using the standard population proposed by Segi (1966) and modified by Doll and Hill [42]. We chose to standardize rates by periods using the direct method to control the effects of age structure on the female population at different periods [43] for each state. It is known that the northeastern states present significant disparities in fertility and mortality rates and, therefore, present important differences in their age structure, justifying the choice of standardization by the direct method.

### Statistical analysis

The effects of age-period-birth cohort (APC) on cervical cancer mortality were estimated for each of the nine states in the Northeast region, considering the Poisson distribution of the number of deaths. The natural logarithm of the expected rate value is a linear function of age, period and cohort effects [25,26].

$$ln(E[r_{ij}])=ln\left(\frac{\theta_{ij}}{N_{ij}}\right)=\mu +\alpha_{i}+\beta_{j}+\gamma_{k},$$

where E[r_ij_] represents the expected mortality rate at age *i* and period *j*; θ_ij_, number of deaths at age *i* and period *j*; N_ij_ denotes the population at risk of death at age *i* and period *j*; μ represents the average rate; α_i_ corresponds to the effect of age group *i*; β_j_, the effect of period *j*; and γ_k_, the effect of cohort *k*.

There is no consensus in the literature about the best methodology to use to correct the complete model identification problem (with the three temporal effects) [26–32] due to the linear relationship existing between these factors. Thus, the APC effect parameters in the present study were estimated using the approach proposed by Holford [26]. This method limits the effect analysis to its linear combinations and curvatures. The curvatures represent estimable functions of the parameters and make them constant, despite the parameterization used [26,27]. In addition, the linear trend of effects is divided into two components: the first is the linear effect of age and the other is called drift, the linear effect of period and cohort [26,27]. The sum of the age and period slopes (α_L_+β_L_) constitutes the longitudinal trend of age, where α_L_ and *β*_*L*_ are linear trend of age and period respectively, whereas the linear trend of the age-specific rates logarithm represents that the drift term is equal to the sum of the period and cohort slopes (β_L_+γ_L_), where β_L_ and *γ*_*L*_ are the linear trend of period and cohort, respectively [26,27].

In the present study, the period from 1995 to 1999 was the reference period, as it corresponded to the previous five-year period and programmatic actions of the Brazilian Ministry of Health for cervical cancer control were specified [2]. The reference cohort was that of 1945–1949, because the central cohorts tend to be more stable and complete than the first and last cohorts [26,27].

The models were compared through the statistics Deviance and the likelihood ratio tests, considering statistically significant results with p ≤ 0.05. The risk of death was estimated by relative risk (RR) estimates and 95% confidence intervals according to period and cohort effects. Estimates for the APC models were made using the Epi library 1.1.18 (R Foundation of Computational Statistics, Vienna, Austria http://www.r-project.org) of the R program (version 3.2.1) [43].

## Results

Over the period investigated, 33,703 deaths in the Northeast region from cervical cancer were reported in women 20 years and older, representing a standardized average rate of 5.31 deaths per 100,000 women. After the procedure to correct the death records, there was a 94.73% increase in cervical cancer deaths (65,630 deaths; 10.35 deaths per 100,000 women) compared to the *SIM/DATASUS-*initially coded records (Table 1).

**Table 1:** Cervical Cancer mortality standardized rates (per 100,000 women) from 1980 to 2014 in the states of northeastern Brazil, according to the stages of the death registration correction process from 1980 to 2014.

States		Periods						
	Rates	1980–84	1985–89	1990–94	1995–99	2000–04	2005–09	2010–14
**AL**	**UMR**	9.11	9.47	6.32	4.86	6.72	8.92	8.86
	**MRC23**	17.77	16.15	13.70	11.21	14.38	13.56	12.60
	**MRC234**	18.35	16.83	14.25	14.06	15.24	14.59	13.49
	**ASC**	18.70	17.16	14.84	14.64	17.38	16.64	15.38
**BA**	**UMR**	5.36	3.32	5.08	5.46	6.03	6.67	7.08
	**MRC23**	10.08	10.60	8.98	9.97	9.35	10.04	10.24
	**MRC234**	10.58	11.04	9.41	10.15	10.31	10.66	10.96
	**ASC**	11.63	12.14	10.83	10.90	10.93	11.73	12.05
**CE**	**UMR**	4.59	3.89	4.54	5.28	7.69	9.51	8.59
	**MRC23**	8.50	8.99	9.60	11.45	13.16	12.88	11.45
	**MRC234**	8.87	9.60	10.13	12.01	14.21	14.06	12.21
	**ASC**	11.53	12.48	12.05	14.31	16.20	15.80	13.92
**MA**	**UMR**	5.28	5.52	5.54	7.52	8.64	14.33	15.22
	**MRC23**	10.21	10.23	11.07	12.63	14.49	19.15	18.41
	**MRC234**	10.73	11.73	11.57	13.62	15.16	20.05	19.38
	**ASC**	20.39	22.29	17.59	23.27	23.68	32.07	31.01
**PB**	**UMR**	3.48	4.40	3.50	2.81	4.19	7.69	7.90
	**MRC23**	10.56	11.00	8.95	7.10	7.73	11.11	11.31
	**MRC234**	11.48	11.53	9.46	12.90	8.34	11.97	12.10
	**ASC**	11.96	12.01	10.06	14.00	9.34	13.41	13.55
**PE**	**UMR**	8.31	6.69	6.79	9.36	10.62	10.87	8.52
	**MRC23**	17.20	12.48	12.33	15.88	16.93	16.17	11.81
	**MRC234**	17.76	13.02	12.66	16.56	17.93	17.18	12.52
	**ASC**	17.76	13.02	12.91	16.90	18.88	18.08	13.18
**PI**	**UMR**	6.68	6.89	4.44	5.50	9.46	11.47	11.49
	**MRC23**	11.39	13.15	8.58	9.15	14.11	14.32	13.98
	**MRC234**	11.80	13.68	8.88	9.56	14.86	14.89	14.72
	**ASC**	21.13	24.50	10.57	11.38	17.28	17.32	17.11
**RN**	**UMR**	7.74	7.16	8.10	6.79	6.96	6.75	5.77
	**MRC23**	13.24	13.76	13.71	12.35	11.64	9.90	7.76
	**MRC234**	13.75	14.25	14.43	13.03	12.50	10.73	8.42
	**ASC**	14.98	15.54	16.16	14.63	14.54	12.47	9.79
**SE**	**UMR**	9.89	8.08	8.75	7.29	10.24	10.48	9.85
	**MRC23**	18.59	16.66	15.90	12.77	15.88	11.74	14.25
	**MRC234**	20.51	17.03	16.54	13.52	16.64	13.12	15.14
	**ASC**	20.92	17.37	17.53	14.37	18.28	14.42	16.63

Uncorrected mortality rates (UMR); Death correction steps 2 and 3: Ill-defined causes+ Unspecified portion of ill-defined uterine cancer-corrected mortality rates (MRC23); Death correction steps 2, 3 and 4: Ill-defined cause-corrected mortality rates, unspecified uterine cancer, and incomplete cancer diagnosis (MRC234); All steps of correction of mortality rates were corrected for ill-defined causes, unspecified uterine cancer, incomplete cancer and underreporting of death (ASC).

After the correction process, there was an increase of more than 60.00% in mortality rates for this cancer in all states of the region, ranging from 68.34% in Sergipe to 137.30% in Maranhão. The significant increase in mortality after all correction stages is due to a higher proportion of deaths classified as nonspecific cervical cancer and lower coverage of death records, especially in the state of Maranhão (Table 1, S2–S4 Figs).

There were no patterns in the data or records missing in elderly. However, worse quality of certification and coverage of death records was observed in the states of Maranhão and Piauí, and in the 1980s and 1990s for all states of the Northeast (Table 1).

There was an increase in five-year mortality rates in the period studied between the first (1980–1984) and the last (2010–2014) in the states of Bahia (3.61%), Ceará (20.72%), Maranhão (52.18%) and Paraíba (13.25%). In other states, there was a reduction in the percentage change between the same periods. In the states of Rio Grande do Norte (-34.64%) and Pernambuco (-25.78%), there were larger percentage reductions between the rates of the first and last five years (Table 1).

The highest standardized average mortality rates per 100,000 women were observed in the states of Maranhão (24.39), Piauí (16.04) and Sergipe (15.69), and the lowest were observed in Bahia (11.24), Paraíba (11.44) and Rio Grande do Norte (12.58).

**Fig 1:**
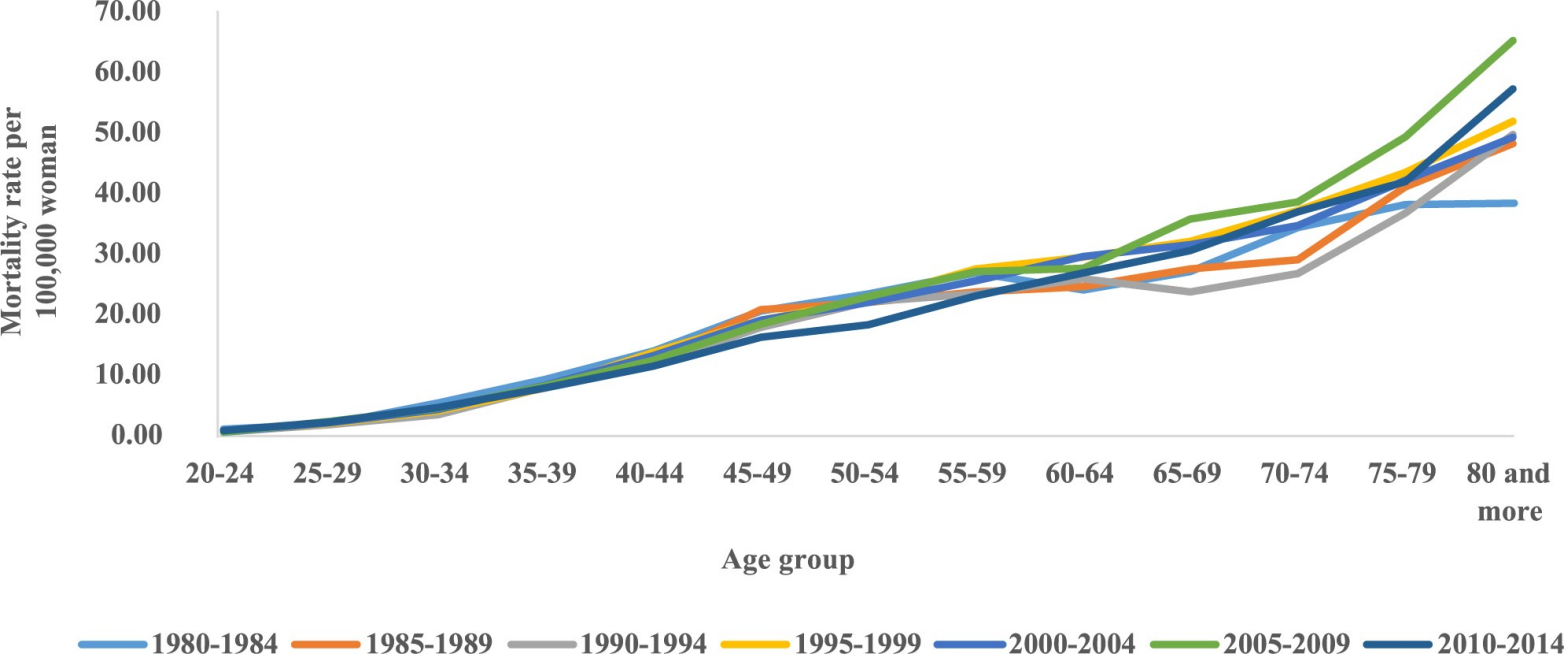
Cervical cancer mortality rates by age group and death period in Northeast Brazil, 1980–2014.

**Fig 2:**
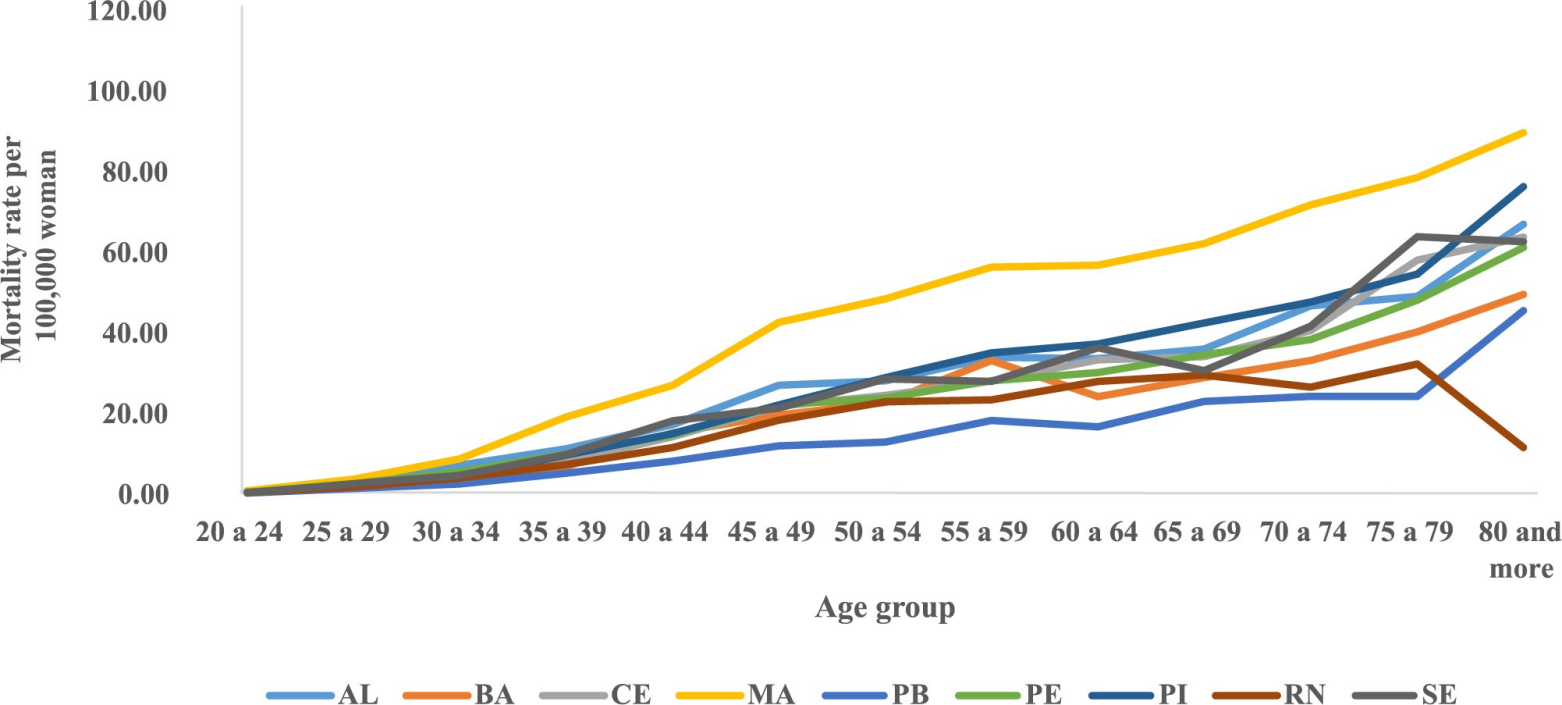
Distribution of mean mortality rates observed for cervical cancer according to age groups in Northeast States, Brazil, 1980–2014.

**Fig 3:**
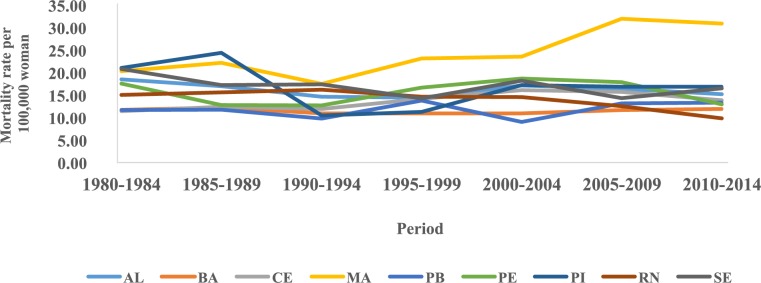
Mortality rates for cervical cancer in the Northeast region of Brazil, by period and age group, 1980 to 2014.

The trend temporal of mortality rates by age group and period in the Northeast region showed a considerable increase in rates from the sixth decade of life in all five-year periods, with the highest rates observed in these age groups in the 2005–2009 period (Fig 1). The evaluation of average mortality rates by age group represents a progressive increase of these coefficients with advancing age, except in the states of Rio Grande do Norte and Sergipe, where there was a reduction in mortality in the age group of 80 years or older. The highest rates were observed in Maranhão and the lowest in Paraíba (Fig 2). In the northeast region of Brazil, mortality rates for all age groups increased in the initial five years of the 2000s, with the highest rates from 2005 to 2009 (Fig 3).

**Fig 4:**
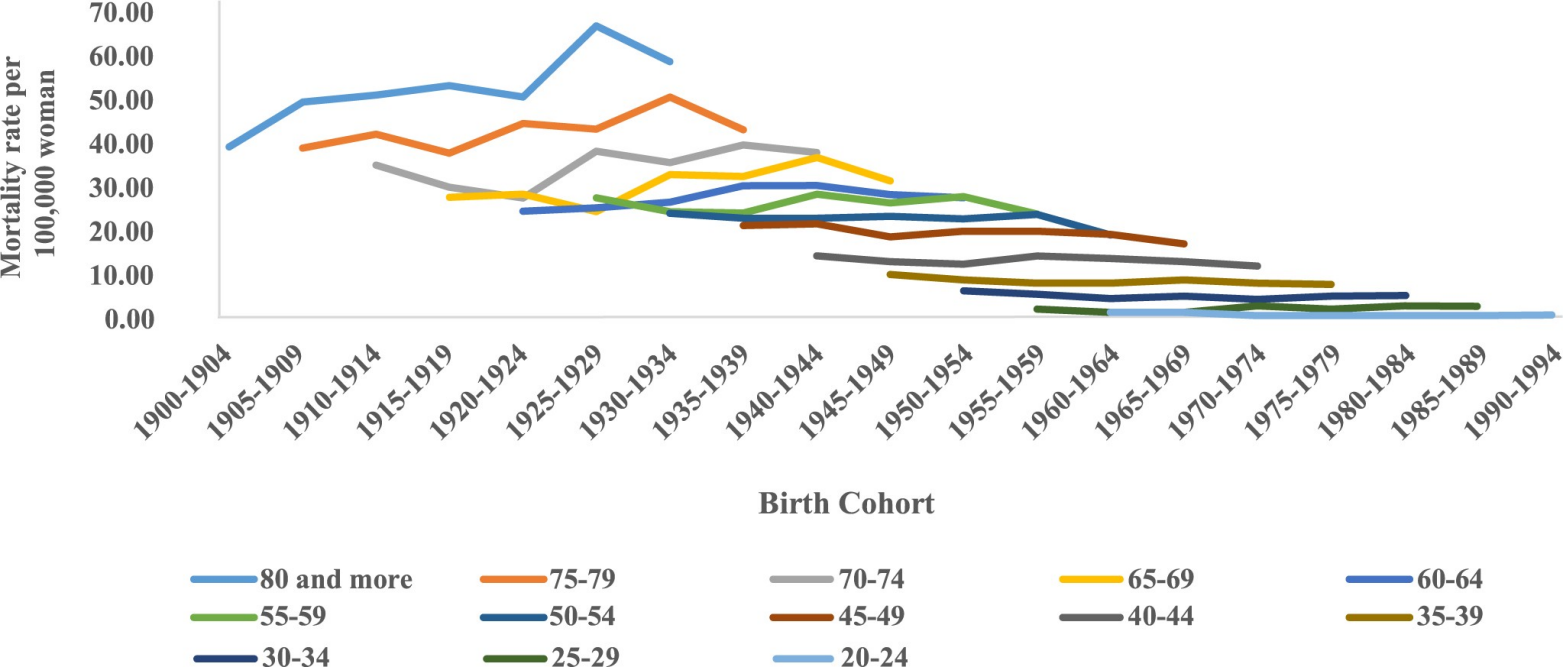
Corrected mortality rates for cervical cancer according to birth cohort and age group in northeastern Brazil, 1980–2014.

**Fig 5:**
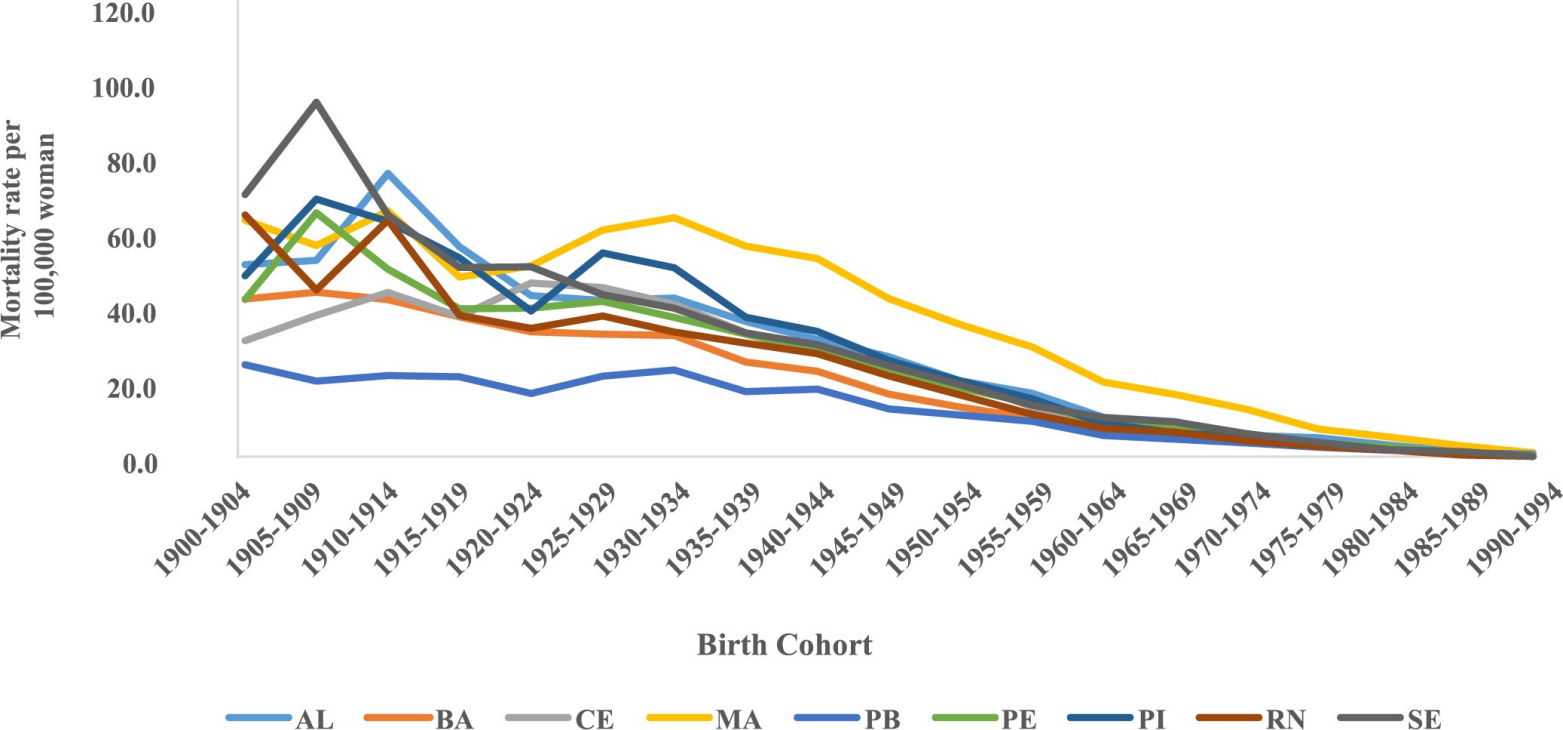
Average mortality rates for cervical cancer according to birth cohort, Northeastern states, Brazil, 1980–2014.

The analysis of mortality by birth cohort showed a reduction in the cohorts from 1925–1929 and in the age group of 80 years and older (Fig 4). Similarly, there was a reduction in average mortality rates in younger cohorts in all states of this region (Fig 5).

**Fig 6:**
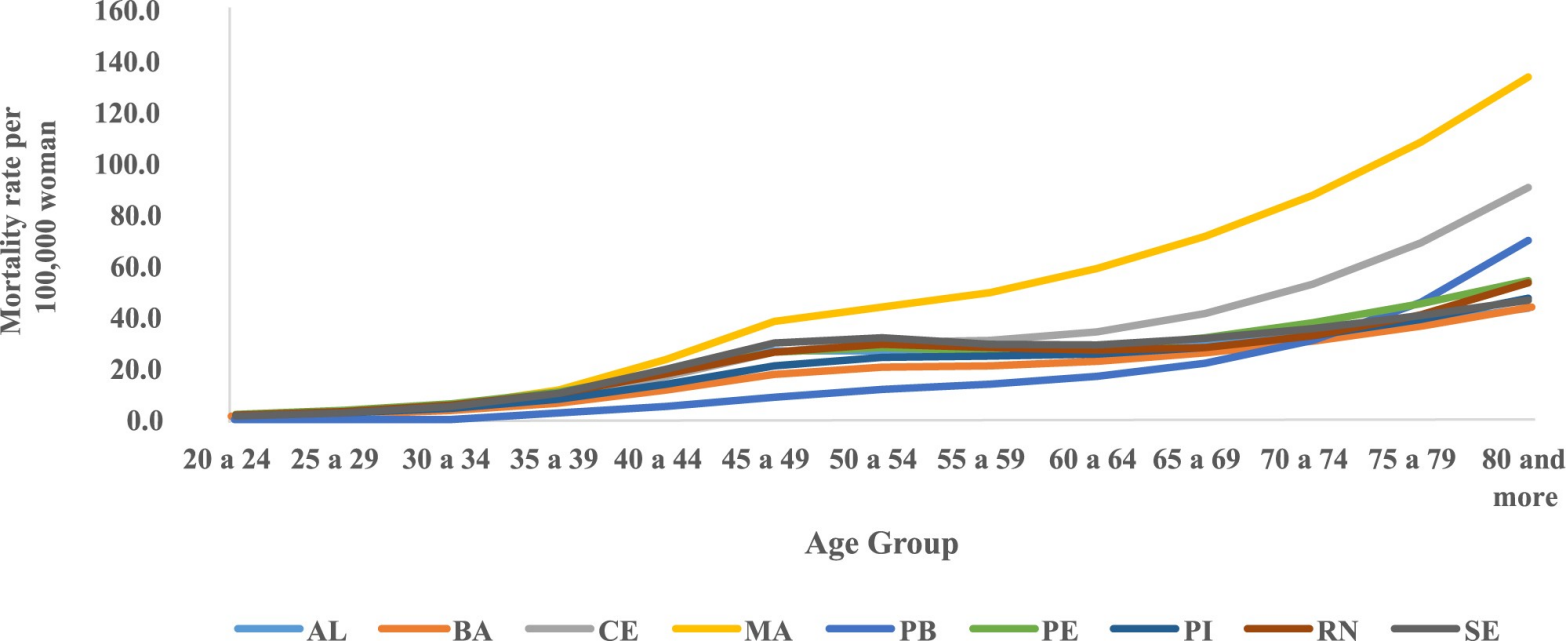
Results of the age-period-cohort model adjusted for cervical cancer mortality according to the age effect and states of the Northeast, Brazil, 1980–2014.

After fitted probabilistic models estimating the effects of age-period and birth cohort, it was found that the complete model presented the best fit to data from all states analyzed (Table 2). Regarding the effect of age, mortality rates increased progressively with advancing age in all Northeastern states, with greater magnitude in Maranhão, from the age group 45–49 years(Fig 6).

**Table 2:** Deviance analysis in sequential construction of APC models.

State of Brazil	Models	DF^a^	Deviance Residual	Pr (>Chi)
AL	Age	86	296.32	
	Age-drift^b^	85	251.37	<0.00001
	Age-Cohort	82	249.5	0.5984
	Age-Period- Cohort	79	213.7	<0.00001
	Age-Period	82	216.62	0.4028
	Age-drift^c^	85	251.37	<0.00001
BA	Age	86	317.09	
	Age-drift^b^	85	304.56	0.0004005
	Age-Cohort	82	273.83	<0.00001
	Age-Period- Cohort	79	268.9	<0.00001
	Age-Period	82	299.24	<0.00001
	Age-drift^c^	85	304.56	0.1494165
CE	Age	86	692.48	
	Age-drift^b^	85	691.1	0.2406
	Age-Cohort	82	534.15	<0.00001
	Age-Period- Cohort	79	346.56	<0.00001
	Age-Period	82	456.96	<0.00001
	Age-drift^c^	85	691.1	<0.00001
MA	Age	86	893.27	
	Age-drift^b^	85	578.94	<0.00001
	Age-Cohort	82	546.75	<0.00001
	Age-Period- Cohort	79	479.4	<0.00001
	Age-Period	82	519.02	<0.00001
	Age-drift^c^	85	578.94	<0.00001
PB	Age	86	537.68	
	Age-drift^b^	85	296.03	<0.00001
	Age-Cohort	82	285	0.011563
	Age-Period- Cohort	79	212.69	<0.00001
	Age-Period	82	228.71	0.001122
	Age-drift^c^	85	296.03	<0.00001
PE	Age	86	537.04	
	Age-drift^b^	85	439.21	<0.00001
	Age-Cohort	82	388.87	<0.00001
	Age-Period- Cohort	79	350.12	<0.00001
	Age-Period	82	392.03	<0.00001
	Age-drift^c^	85	439.21	<0.00001
PI	Age	86	508.34	
	Age-drift^b^	85	482.16	<0.00001
	Age-Cohort	82	461.26	<0.00001
	Age-Period- Cohort	79	319.3	<0.00001
	Age-Period	82	357.86	<0.00001
	Age-drift^c^	85	482.16	<0.00001
RN	Age	86	341.79	
	Age-drift^b^	85	244.21	<0.00001
	Age-Cohort	82	207.72	<0.00001
	Age-Period- Cohort	79	177.9	<0.00001
	Age-Period	82	205.72	<0.00001
	Age-drift^c^	85	244.21	<0.00001
SE	Age	86	242.28	
	Age-drift^b^	85	195.87	<0.00001
	Age-Cohort	82	193.88	0.5746256
	Age-Period- Cohort	79	173.93	0.0001744
	Age-Period	82	178.99	0.1677603
	Age-drift^c^	85	195.87	0.0007491

^a^Degrees of freedom

^b^linear trend of the logarithm of age-specific rates, which is equal to the sum of the of period and cohort slopes (βL + γL), where βL and γL are the linear trends for the period and cohort, respectively.

^c^longitudinal trend of age is the sum of age and period slopes (αL + βL), where αL and βL are the linear trends of age and period, respectively.

**Fig 7:**
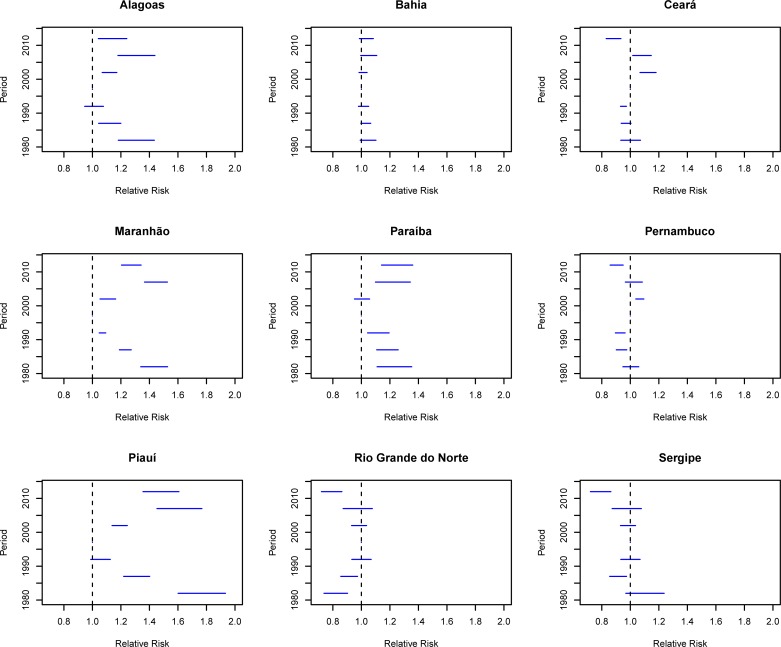
Results of the age-period-cohort model adjusted for cervical cancer mortality according to the period effect and states of the Northeast, Brazil, 1980–2014.

Assessing the period effect adjusted for the effects of age and birth cohort revealed an increased risk of death (RR> 1) for cervical cancer in all five-years of the 2000s compared with the reference period (1995–1999) in the states of Alagoas, Maranhão and Piauí. In Paraíba, there was an increase in the period from 2005 to 2014, while in Ceará, the increase occurred in the period from 2000 to 2009, with simultaneous reduction of the risk (Fig 7).

In Pernambuco, there was an increase only from 2000 to 2004, for the next five-years, the increase was not statistically significant (RR = 1.03, CI 95% 0.97–1.08). The state of Bahia had an increased risk of death, but was not statistically significant in any of the analyzed periods (Fig 7). However, in Sergipe and Rio Grande do Norte there has been a reduction in the risk of death in the last five years (2010–2014) (Fig 7).

**Fig 8:**
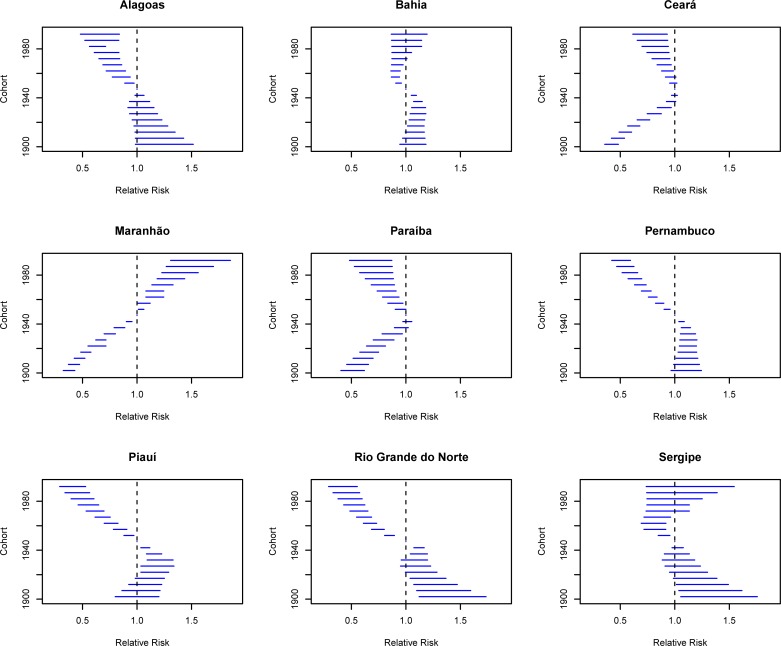
Results of the age-period-cohort model adjusted for cervical cancer mortality according to the cohort effect in northeastern states, Brazil, 1980–2014.

Regarding the effect of birth cohort, there was a progressive reduction of risk in younger cohorts between 1945 and1949 in states of Alagoas, Bahia, Ceará, Pernambuco, Piauí, Rio Grande do Norte and Sergipe (Fig 8). In the states of Bahia, Rio Grande do Norte and Sergipe, this reduction was statistically significant only for the cohorts from 1950 to 1969. However, in Maranhão, there was an increased risk of death for women born after 1950 (Fig 8). In Paraíba, all cohorts had a risk below one (RR<1) when compared to the 1945–1949 cohort.

## Discussion

The quality and coverage of information systems play important roles in the temporal trend of incidence and mortality of health problems [25,26]. Inequalities in access to health services are correlated with poor coverage and inadequate certification of the underlying cause of death [25,26].

In this study, we chose to correct the death records due to ill-defined causes, unspecified uterine cancer and incomplete diagnosis of general cancer and female genital tract, in order to obtain more reliable mortality rates for cervical cancer. As there were significant improvements in coverage and quality of death records in the Northeast region of Brazil in the 2000s, this may influence conclusions about the temporal trend and APC effects on cervical cancer mortality [37–38].

The increased access to health services associated with diagnostic innovations correlates with better coverage and quality of death records in all age groups, which may influence the temporal trend of mortality. With regard to cervical cancer mortality, improvements in the quality and coverage of Mortality Information System may increase deaths from this cancer as there is a reduction in records classified as an ill-defined cause, incomplete diagnosis of general cancer, diagnosis of incomplete cancer for female genital tract, unspecified portion of uterus cancer. Thus, the observed increase in mortality rates after expanding access to health services may correlate with better quality of death information rather than with increased incidence rates and reduced survival [20,39–41].

This hypothesis was corroborated by the results presented in S4 and S5 Figs, comparing the effects of period and birth cohort for uncorrected cervical cancer death records and those corrected by the indirect technique.

After the correction process, there was a significant increase in deaths in states with the worst socioeconomic conditions (Maranhão and Piauí) and in the 1980s and 1990s. Thus, when evaluating the period effect comparing corrected and uncorrected data, higher relative risk (RR) values in the 2000s compared with 1995–1999 in cervical cancer mortality with uncorrected records were observed. Highlighting the states of Alagoas, Ceará, Paraíba, Piauí (2000 to 2009).

Furtheremore, it was observed that in the uncorrected data, women of younger generations, compared to the reference cohort (1945–1949), had a higher risk of death than older cohorts. The opposite occurred with corrected data.

We believe that the differences observed in the temporal trend in corrected and uncorrected data are possibly related to improvements in the quality of information presented by death records in Brazilian states in the 2000s [19,39–41]. Due to the expansion of access to health services after the implementation of the Unified Health System (Sistema Único de Saúde)[19,39–41].

Therefore, women who died in the 2000s, especially those from younger cohorts, are more likely to have their underlying cause of death ranked correctly when compared to women who died in the 1980s and 1990s. Thus, without the use of indirect correction techniques, we could raise wrong assumptions about the effects of age, period, and cohort on cervical cancer mortality [19,39–41].

From this perspective, a study by Gamarra et al. [19] in the states of northeastern Brazil from 1996 to 2005 showed the significant increase in cervical cancer mortality rates after the correction processes in the inner cities. Prior to the correction process, higher mortality and upward trend in cities in the Northeast with the best socioeconomic conditions and lower mortality rates and downward trend in cities with the worst socioeconomic conditions. However, an inverse profile was verified after the correction process. This reality is correlated with disparity in access to health care, and therefore women living in poorer locations in Brazil are less likely to diagnose their disease, contributing to poor quality of *SIM/DATASUS* data information [19,37–38].

This study stands out because the northeastern states presented higher mortality rates for cervical cancer in all five-year periods (1980–1984 to 2010–2014) compared to places with greater socioeconomic development and better access to health services [1,10,20].

After the correction stages, all states had rates of more than 9.0 deaths per 100,000 women, a much higher mortality rate than that of the Brazilian states with the highest socioeconomic development (4.0 deaths per 100,000 women) [18] and by local with organized screening programs such as North America (1.9 deaths per 100,000 women) and Northern Europe (2.1 deaths per 100,000 women) [1]. However, it was similar to mortality rates in regions with low socioeconomic development, such as the Caribbean (8.5 deaths per 100,000 women) and South America (7.1 deaths per 100,000 women) [1].

In this scenario, the states of Maranhão, Sergipe, Piauí and Alagoas can be highlighted with higher mortality rates in the Northeast of Brazil, confirming the results observed from 1996 to 2005 [19]. These states have a similar mortality rate from cervical cancer to that observed in regions where there is no universal health system and screening program. It is also noted that these states are the areas of greatest socioeconomic vulnerability in Brazil and have the worst health indicators in the Northeast [19–20,34].

These results were expected and are explained by the transition theory to cancer, correlated with the epidemiological transition, described as having a direct relationship with the Human Development Index (HDI) [1,10]. Thus, higher rates of cancer associated with infection were observed in places with lower socioeconomic development, indicating difficulty in accessing health in these regions. This contrasts with countries with higher socioeconomic development, where an increase in cancer is associated with westernized habits and lifestyles [1,10].

A transition to cancer has been confirmed by some studies in Brazil [17–18,35,44]. There is an upward temporal trend in stomach cancer mortality in the North and Northeast regions, and a downward trend in more developed regions [44]; however, a positive correlation is observed between the human development index (HDI) value and breast cancer mortality, while there was a negative correlation between this indicator and cervical cancer mortality [20,36].

In the present study, we aimed to evaluate if the existence of a universal and open access cancer care network, which includes early detection (Pap Test), the treatment of cervical cancer promoted a period effect, reducing mortality by cervical cancer, especially in the last two quinquennials analyzed (2005–2009 and 2010–2014) and in younger cohorts with greater screening coverage, as well as greater access to health services for cancer treatment [14,16–17].

Thus, the use of APC models is justified because the analysis of the temporal trend of diseases according to the effects of age, period and cohort is an important tool to raise hypotheses about the efficiency and effectiveness of health policies and programs (period effect), as well as assessing the correlation of increased prevalence of risk and protective factors for incidence and mortality rates of diseases and health problems [26,27].

Regarding the effect of age, there was an increase in cervical cancer mortality with advancing age groups in the Northeast region, which was an expected finding, as it is a chronic disease in which the risk of disease and death increases with exposure to life-long risk factors.

Moreover, at the end of the reproductive period, women have less frequent gynecological consultations, with the lowest coverage of preventive examinations in all Brazilian regions [14,16–17], increasing the likelihood that older women will be diagnosed with advanced disease when compared to younger women. Corroborating this hypothesis, in Brazil, from 2000 to 2012, there was a progressive increase in the chance of advanced disease in the diagnosis with advancing age, the highest odds ratio was presented in women 70 years and older (OR = 2.24; 95% CI 1.87–2.69) when compared to those under 30 years old [45].

A decade after the implementation of the Cervical Cancer Prevention and Treatment Program, mortality rates and risk cervical cancer death were expected to decrease over the last two five-year periods of the historic series analyzed (2005–2009 and 2010–2014).

However, in the Northeast, the states of Bahia, Ceará, Maranhão and Paraíba presented positive percentage variation when comparing the first (1980–1984) and the last five years of the historical series (2010–2014). In the five-year periods of the 2000s, there was an increased risk of death from cervical cancer in the states of Alagoas, Maranhão, Paraíba and Piauí (RR> 1, p ≤0.05), and only in the states of Ceará, Pernambuco and Rio Grande do Norte there was a reduction in the risk of death from this cancer in the period 2010–2014 (RR<1, p≤0.05).

In the early years of the screening program implementation, an increase in cervical cancer incidence and mortality is expected, because many women can be diagnosed in advanced stages of the disease by accessing the Pap Test. However, the existence of a good quality screening program with good coverage may influence the reduction of the mortality rate over the years, due to the diagnosis and treatment of precursor lesions, decreased incidence of the disease and increased survival due to the timely diagnosis and cervical cancer treatment [3–11].

It is also necessary to consider the impact of therapeutic innovations on the temporal trend of cervical cancer mortality, such as the emergency of the oncologic gynecology specialization, the introduction of radiotherapy treatment and its subsequent association with platinum-based chemotherapy [3–11]. However, major changes in the treatment of advanced cervical cancer have not been observed since the 1990s, and therefore the reduction in mortality over the last two decades in many countries has been correlated with the effectiveness of screening programs [3,11].

The cervical cancer screening program has as its limitation the possibility of detecting abnormalities that would never become clinically apparent in the absence of Pap test, as cervical cancer precursor epithelial lesions may regress spontaneously. [46–49]. The detection of such abnormalities is called overdiagnosis because diagnostic lesions or diseases that could never cause symptoms or death in the individual's life are detected [46–49]. Often overdiagnosis can lead to overtreatment. In this sense, a study developed using simulation techniques showed a high frequency of overdiagnosis in the Dutch screening program, representing 70% of overdiagnosis when evaluating all stages of cervical intraepithelial neoplasia (CIN) and invasive cancer. [48].

According to Van Luijt et al (2016), the impact of overdiagnosis depends on its frequency in the population undergoing the screening program and the extent of the proposed treatment with regard to the invasiveness, costs and acceptability of the population. Thus, these authors argue that while the frequency of overdiagnosis was higher in cervical cancer screening compared to breast cancer screening, its impact is more limited. As the treatment of cervical cancer precursor lesions is minimally invasive and is performed on an outpatient basis (biopsy and conization by high-frequency surgery) [48].

However, although this procedure is of limited risk, overtreatment may promote: (i) financial impacts on health systems; (ii) contribute to the delay between referral and treatment as many women are undergoing biopsy and unnecessary care; and (iii) may affect the mental health of women who were diagnosed with the precursor lesion and underwent biopsy and conization [46–49]. Thus, to reduce the impact of overdiagnosis and overtreatment, research in molecular and cellular biology to differentiate cancer and indolent lesions from those with very aggressive potential is suggested [46–49]. Despite the existence of this limitation, cervical cancer screening is an important tool in the prevention and control of this cancer [3,11,46–49].

In the present study, the increased risk of death in the 2000s for most northeastern states correlates with low quality and low PNCC coverage, as barriers to accessing the cancer care network.

These results differ from those observed in the municipalities of São Paulo and Rio de Janeiro from 1980 to 2009, when a reduction in the risk of cervical cancer death was observed in the five-year periods of the 2000s (2000–2004 and 2005–2009) [13]. Women living in these municipalities have the highest coverage of Pap Test in Brazil [16–17,22,50–51]. In addition, these municipalities have had a cervical cancer screening program since the 1980s [2] and have the highest concentration of the Brazilian cancer care network. However, these time trends and period effects are similar to those observed in Western Europe countries, the Baltic countries, and Central Asia regions that have implemented recent low coverage screening programs [9,11].

In fact, the Brazilian Northeast is a region with low socioeconomic development and difficulties in access to health services, a scenario that remains in the 2000s, even after the beginning of public policies of income redistribution and the expansion of access to health care through the Family Health Strategy. In this location, compared to other Brazilian regions, there is a worse self-rated health condition, lower use of health service [14], and lower performance coverage of Pap Tests [16–17].

This conjuncture contributes to the high mortality rates observed in the present study, especially after the correction process of deaths classified as unspecified uterine cancer, and death coverage in Maranhão and Piauí. This reflects the low effectiveness and efficiency of the health system in these regions, as a large number of women continue to be diagnosed at the advanced stage of CC, or without identifying the exact uterine site at which the disease began [20–22]. In addition, many deaths from CC are not yet registered in the official death system due to underreporting of death records [40].

This pattern is supposed to be maintained in Brazil due to the characteristics of the screening program (PNCC), which allows some women to perform more tests than recommended, while others never do them, especially low-educated,black women than living in regions with greater social vulnerability [18–20,52–55]. Another issue that deserves attention in the *PNCC* is the quality of the exams collected, as there is still a high proportion of unsatisfactory samples, sample rejection and lack of transformation zone (TZ) in the sample. In addition, inconsistency with that recommended by the Ministry of Health regarding the positivity index indicates significant problems in the collection, storage and reading of samples/slides [52,55].

In addition to the low coverage and poor quality of screening programs, the PNCC presents difficulties associated with the referral of women with cervical cancer or their precursor lesions to treatment in specialized services (surgery, chemotherapy and radiotherapy) [56–59], correlating with the large proportion of women diagnosed in advanced stages of this disease (III/IV). In Brazil, from 2006 to 2012, only 29% of women with CC were diagnosed at the early stage of the disease (in situ or stage IA carcinoma). From 2000 to 2012, there was an increase of 1.10% per year in the proportion of women diagnosed at an advanced stage [45,59].

The presence of an advanced tumor (lymph node involvement and the presence of distant metastases) in the diagnosis represents the main factor associated with shorter five-year survival in women diagnosed with cervical cancer [56–58]. Women in group I (stages IA to IB) had a five-year survival of 92.3%, while women in group II (stages IIA to IVB) had a five-year survival of only 32.7% [58]. Women living in the Northeast were more likely to be diagnosed at an advanced stage than women living in the Southeast (OR = 1.32; 95% CI 1.25–1.40) [45].

Southeast Brazil is a region with great socioeconomic development and the highest concentration of the Brazilian cancer care network [45], while the North and Northeast are the Brazilian regions with the smallest cancer care network and the largest deficits in radiotherapy devices [54] and chemotherapy, services widely used in the treatment of advanced cervical cancer [11,45,55,60].

The cohort mortality risk showed three profiles: an increase was observed for younger generations compared to the reference cohort in Maranhão (1945–1949), while there was protective effect (RR<1) for cervical cancer mortality in women of younger generations in other states; there was protection from all cohorts in Paraíba.

The risk reduction for younger cohorts observed in the states of Alagoas, Bahia, Ceará, Pernambuco, Piauí, Rio Grande do Norte and Sergipe is comparable to that observed in the two Brazilian cities of São Paulo and Rio de Janeiro [13] and countries from Northern Europe, the United Kingdom, Canada, the United States and Singapore [3–10]. Maranhão’s profile is similar to Estonia, Baltic countries and Western Europe [5,61].

Differences in risk patterns of cervical cancer incidence and mortality according to age, period and cohort between different sites may be correlated with the interaction of two key factors, namely, the existence of a prevention program and treatment of cervical cancer (screening program and cancer care network) and change in women’s sexual and reproductive behavior [3–11].

The reduction in risk of death for younger cohorts, which occurred in more than 70% of states in the Northeast region, may be correlated with greater coverage screening coverage, as well as increased access to health services for cancer treatment observed in younger women [10,14,16–17]. Thus, the generation of women influenced by the sexual revolution and behaviors of the 1960s to 1970s, which led to greater exposure to risk factors for cervical cancer by having access to a protective measure (Pap test and cancer care network possibly protected them from disease and death from this cancer) [3–10,61].

This reality may also correlate with the implementation of the Unified Health System in Brazil, which increased acess to health for a significant portion of the population and promoted an increase in primary care services through the Family Health Strategy in the 2000s, with greater coverage of this strategy in places of the country with greater socioeconomic vulnerability [15]. However, it is important to note that despite the reduced risk of death in younger cohorts, the average mortality rates for these generations remained high compared to countries that have a long-term, high coverage and high screening program access to cancer treatment [3–11].

In theory, the implementation of a free universal access prevention and treatment program would have a period effect, because all female age groups of the recommended ages would be exposed to this secondary prevention and treatment measure. However, greater coverage has been observed for younger women since the implementation of the *PNCC*, a condition that is maintained when the analysis was stratified by race/color, education, marital status, and place of residence [14,16–17,21,62]. Similarly, greater access to health services and cancer care network is observed in younger women [14–16].

Therefore, differences in coverage of participation the observed in screening programs and access to health services by age group would could promote a cohort effect on cervical cancer incidence and mortality, considering that exposure to these factors of protection is differentiated according to age [17,22,46,50,63].

Findings in Paraíba, where all cohorts are at risk of death and reduced risk of death from cervical cancer compared to the reference cohort (1945–1949), may be related to the sexual and reproductive behaviors of resident women in this place. This behavior may reduce the risk of death from this cancer, even for women of older generations not exposed to protective factors (screening program and cancer care network) [3,11], a hypothesis that deserves to be studied in future research.

These results are equivalent to those observed in Spain (1951–1991) and Shandong in China (1970–1992), because the authors argue that the decreasing temporal trend and the incidence and mortality of cervical cancer may be related to the decreasing prevalence of risk factors associated with sexual and reproductive behavior, because at the time there was no free screening and treatment program with universal access in these countries [64–65].

In China, the authors state that reducing the risk of death of cervical cancer death, especially in younger cohorts, was probabily related to the prohibition of prostitution and the institution of the one-child policy [66]. Similary, in Spain, the lower magnitude of mortality rates from this cancer until 1975 was correlated with: a large proportion of women who started their sex life at an older age; reduced number of sexual partners; a late diffusion of hormonal contraception [66].

A possible explanation for the increased risk of death in younger cohorts in the state of Maranhão includes limited access to quality Pap tests and reduced cancer care for women suffering in state in the northeast. Younger women are more vulnerable and suffer from cervical cancer due to greater exposure to risk factors for this disease [2,10]. Another hypothesis may be the change in histological type in younger women, which occurred in South Korea, for example, where high mortality in younger generations was correlated with an increased incidence of adenocarcinoma [63].

This type of histology is more difficult to diagnose through oncotic cytology due to its location in the cervical canal and its rapid growth [3,11,58]; This conjecture deserves to be evaluated in future research. However, it is important to note that more than 80% of cervical cancer diagnosed in Brazil between 2000 and 2012 were squamous cell carcinomas [46,64]. In addition, the increased incidence and mortality from adenocarcinoma correlates as the efficiency and effectiveness of the screening program increases [64]. However, this was not the case with the screening program in this northeastern state [3,19–20,55].

Disparities in the quality of information between states in the Northeast region may be a limitation of this study; however, corrections were made, producing more reliable mortality estimates. Moreover, it was not possible to analyze mortality according to histological type. Some studies have shown that although screening programs improve coverage and quality, there is a substitution of a more aggressive histological type from squamous cell carcinoma to adenocarcinoma [3,63].

Another limitation concerns APC models, as there is no consensus in the literature about a better method to correct the problem of full model identification. However, the model was estimated using estimable functions, which is a more recommended methodology in studies comparing classical statistical methods [26,27].

## Conclusion

The present study showed a worrying situation in cervical cancer mortality in the Northeastern states, especially in Maranhão, Sergipe, Alagoas and Piauí. Having a prevention and treatment program for over a decade was not enough to reduce the risk of death in the last five years of the 2000s or to reduce cervical cancer mortality rates. This reality points to problems in the *PNCC* that include low coverage of women aged 25–64 [14,16–17], limited quality of collection, storage, and reading of slides [52,54], as well as difficulties in referral to the specialized care network in cases of precursor injury or cervical cancer [53–54,60].

Based on their findings the authors believe that actions be taken to increase the coverage of Pap Test in women living in the states of northeastern Brazil, especially among black women with low education, as they have the lowest test coverage and the highest risk of disease and death from cervical cancer. It is suggested that the PNCC be revised in order to reorient the practice of screening, moving to organized screening, especially in places with low spontaneous adherence and high coverage by primary health care. In addition, primary care physicians and nurses need to be trained in relation to PNCC guidelines regarding collection, storage, follow-up, and referral to the specialist network in situations where Pap tests show changes [53–54,60].

Associated with the measures described above, it is necessary that the states of Northeastern Brazil evaluate the referral and counter-referral process for the specialized cancer network, especially regarding: (i) time between referral and colposcopy;(ii) time between referral and biopsy; and (iii) the time between diagnosis of cervical cancer and initiation of treatment [66,67]. Since the longer the time taken to access these procedures and postpone timely treatment increases the diagnosis at advanced stages and reduces the survival of these women [45,66].

## Supporting information

S1 Fig Corrected and uncorrected cervical cancer death records in the states of northeastern Brazil from 1980 to 2014. (TIF)

S2 Fig Corrected and uncorrected cervical cancer death records in the states of northeastern Brazil from 1980 to 2014. (TIF)

S3 Fig Corrected and uncorrected cervical cancer mortality rates in the states of northeastern Brazil from 1980 to 2014. (TIF)

S4 Fig Period effects adjusted by the age and birth cohort effects for cervical cancer mortality in the Northeastern Brazilian states, including corrected and uncorrected deaths, from 1980 to 2014. (EPS)

S5 Fig Birth cohort effects adjusted by the age and period effects for cervical cancer mortality in the Northeastern Brazilian states, including corrected and uncorrected deaths, from 1980 to 2014. (EPS)

S1 Table Corrected and uncorrected cervical cancer cervical death records for the Northeast states of Brazil, from 1980 to 2014. (DOCX)
