# Supplementary figures and images for: Analysis of the effects of the age-period-birth cohort on cervical cancer mortality in the Brazilian Northeast
Source: PLoS One. 2020 Feb 19;15(2):e0226258. doi: 10.1371/journal.pone.0226258 (PMC7029866; doi:10.1371/journal.pone.0226258)

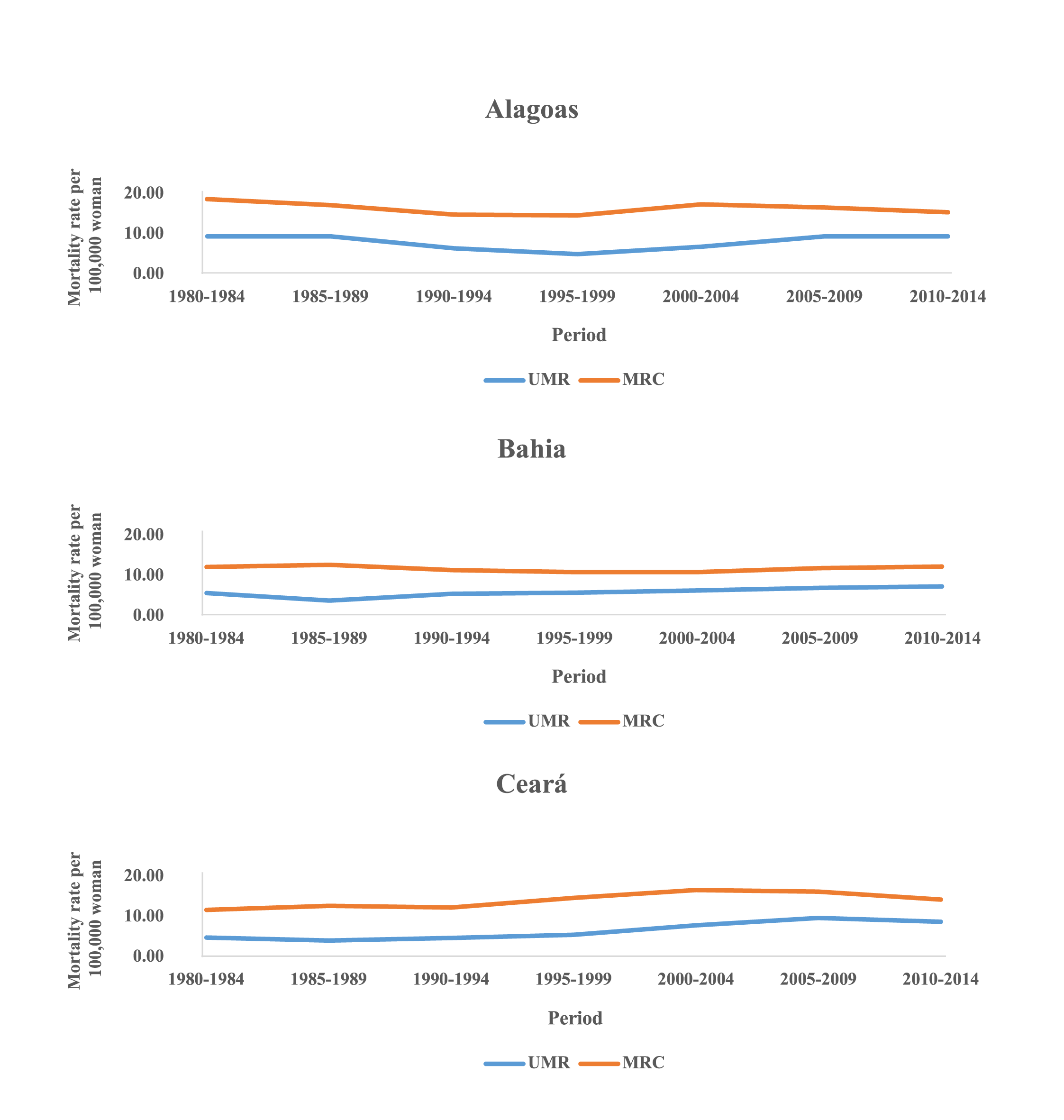

Supplement: S1 Fig — (TIF) [file pone.0226258.s001.tif]

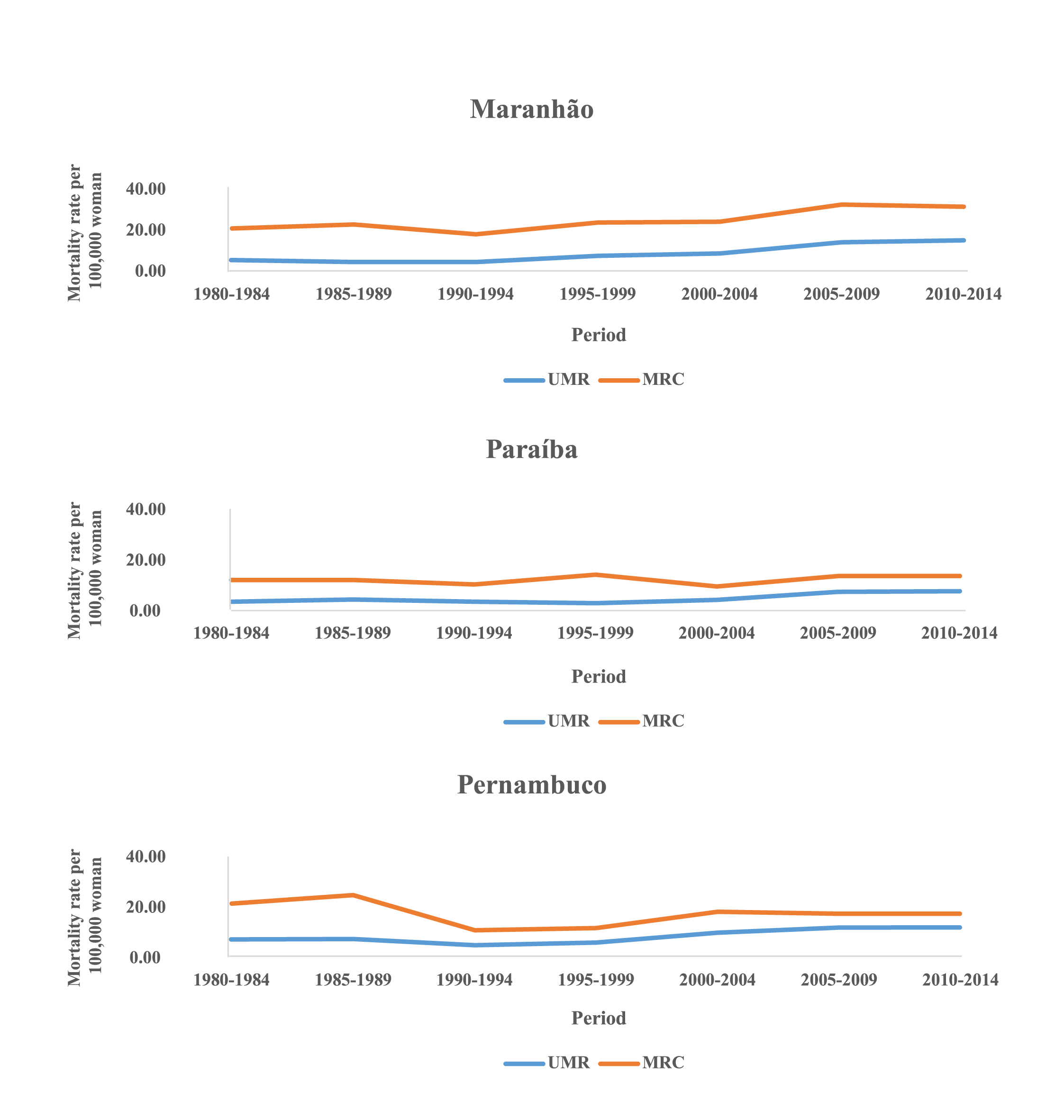

Supplement: S2 Fig — (TIF) [file pone.0226258.s002.tif]

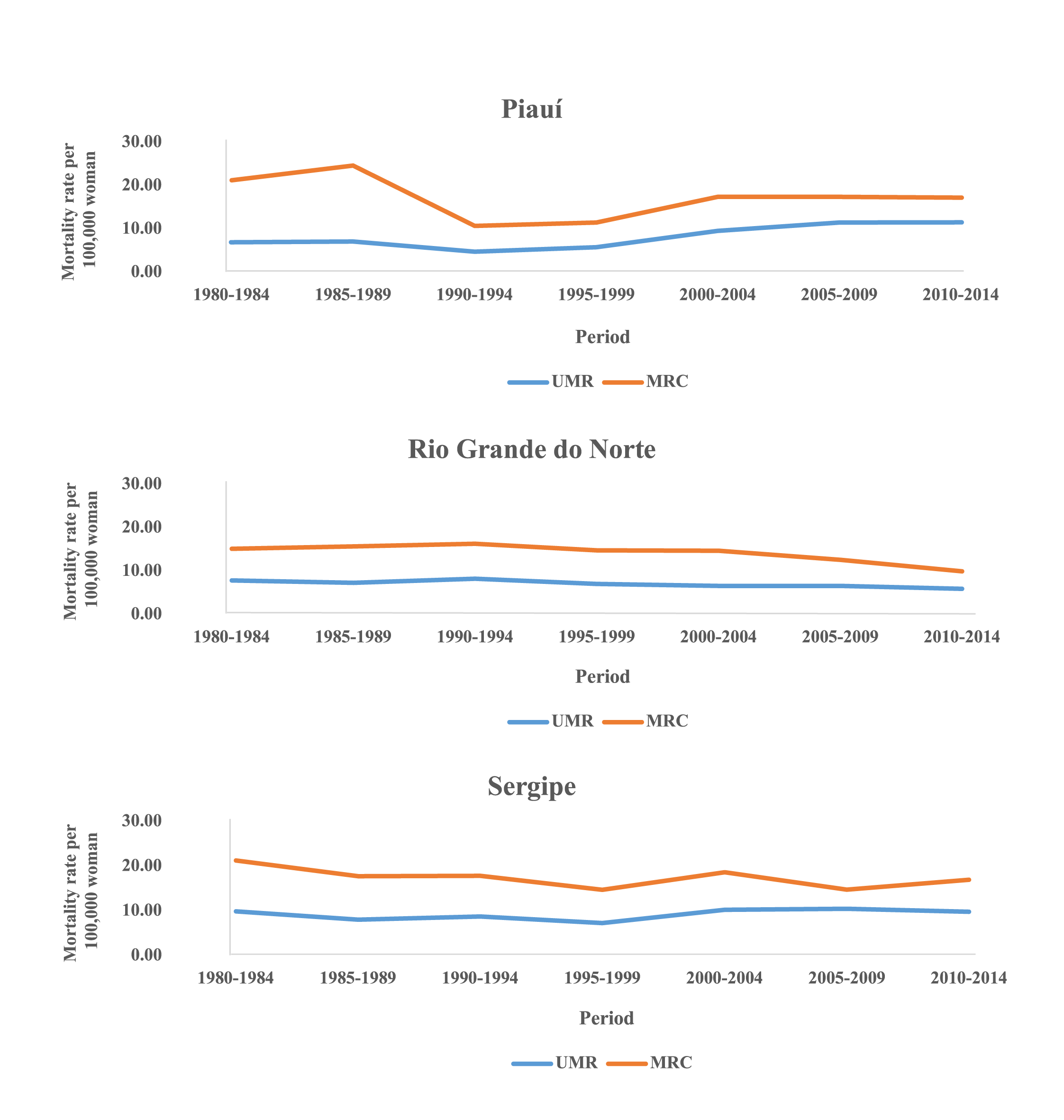

Supplement: S3 Fig — (TIF) [file pone.0226258.s003.tif]
