# Supplementary material for: Analysis of the effects of the age-period-birth cohort on cervical cancer mortality in the Brazilian Northeast
Source: PLoS One. 2020 Feb 19;15(2):e0226258. doi: 10.1371/journal.pone.0226258 (PMC7029866; doi:10.1371/journal.pone.0226258)
Supplement: S1 Table — (DOCX) [file pone.0226258.s006.docx]

|  |  |  |  |  |  |  |  |  |  |  |
| --- | --- | --- | --- | --- | --- | --- | --- | --- | --- | --- |
|  |  |  |  | Uncorrected cervical cancer death records | | | | |  |  |
| Period | AL | BA | CE | MA | PB | PE | PI | RN | SE | NE |
| 1980-1984 | 220 | 624 | 310 | 243 | 124 | 668 | 168 | 192 | 139 | 2688 |
| 1985-1989 | 268 | 765 | 301 | 287 | 176 | 721 | 200 | 210 | 140 | 3068 |
| 1990-1994 | 203 | 777 | 392 | 319 | 154 | 801 | 145 | 271 | 172 | 3234 |
| 1995-1999 | 172 | 937 | 506 | 663 | 134 | 1020 | 196 | 251 | 164 | 4043 |
| 2000-2004 | 271 | 1157 | 857 | 641 | 222 | 1109 | 387 | 297 | 272 | 5213 |
| 2005-2009 | 403 | 1460 | 1222 | 1234 | 453 | 1287 | 529 | 329 | 320 | 7237 |
| 2010-2014 | 458 | 1729 | 1246 | 1539 | 525 | 1329 | 603 | 449 | 342 | 8220 |
| Total | 1995 | 7449 | 4834 | 4926 | 1788 | 6935 | 2228 | 1999 | 1549 | 33703 |
| Cervical cancer death records after the five death correction steps | | | | | | | | | | |
| Period | AL | BA | CE | MA | PB | PE | PI | RN | SE | NE |
| 1980-1984 | 452 | 1292 | 723 | 887 | 386 | 1339 | 482 | 334 | 271 | 6165 |
| 1985-1989 | 486 | 1577 | 909 | 1071 | 426 | 1307 | 651 | 417 | 273 | 7116 |
| 1990-1994 | 464 | 1606 | 972 | 970 | 407 | 1437 | 326 | 507 | 299 | 6987 |
| 1995-1999 | 421 | 1870 | 1327 | 1278 | 592 | 1767 | 383 | 508 | 299 | 8444 |
| 2000-2004 | 666 | 2099 | 1760 | 1680 | 465 | 1896 | 673 | 583 | 465 | 10286 |
| 2005-2009 | 744 | 2513 | 2031 | 2739 | 782 | 2099 | 798 | 598 | 432 | 12736 |
| 2010-2014 | 785 | 2882 | 1993 | 3108 | 898 | 2023 | 887 | 750 | 570 | 13896 |
| Total | 4016 | 13839 | 9716 | 11731 | 3955 | 11867 | 4202 | 3697 | 2608 | 65630 |
